# Proteomic Technologies for the Study of Osteosarcoma

**DOI:** 10.1155/2012/169416

**Published:** 2012-03-06

**Authors:** Stephanie D. Byrum, Charity L. Washam, Corey O. Montgomery, Alan J. Tackett, Larry J. Suva

**Affiliations:** ^1^Department of Biochemistry and Molecular Biology, University of Arkansas for Medical Sciences, 4301 West Markham, Little Rock, AR 72205, USA; ^2^Department of Orthopedic Surgery, Center for Orthopedic Research, Winthrop P. Rockefeller Cancer Institute, University of Arkansas for Medical Sciences, 4301 West Markham, Little Rock, AR 72205, USA

## Abstract

Osteosarcoma is the most common primary bone cancer of children and is established during stages of rapid bone growth. The disease is a consequence of immature osteoblast differentiation, which gives way to a rapidly synthesized incompletely mineralized and disorganized bone matrix. The mechanism of osteosarcoma tumorogenesis is poorly understood, and few proteomic studies have been used to interrogate the disease thus far. Accordingly, these studies have identified proteins that have been known to be associated with other malignancies, rather than being osteosarcoma specific. In this paper, we focus on the growing list of available state-of-the-art proteomic technologies and their specific application to the discovery of novel osteosarcoma diagnostic and therapeutic targets. The current signaling markers/pathways associated with primary and metastatic osteosarcoma that have been identified by early-stage proteomic technologies thus far are also described.

## 1. Introduction

Osteosarcoma (OS) accounts for approximately 3% of all childhood cancers [1]. The majority of osteosarcoma cases arise in children and young adults between 10 and 30 years of age during the years of bone development [1]. Currently, patients diagnosed with localized OS have a 5-year survival rate between 60 and 80%; however, in those patients with metastases present at the time of diagnosis the 5-year survival rate falls dramatically to between 15 and 30% [1]. The current treatment options for patients with primary OS include chemotherapy and surgical removal [1]. Osteosarcoma preferentially metastasizes to the lungs resulting in respiratory failure and patient death if treatments are ineffective [2, 3].

**Figure 1:**
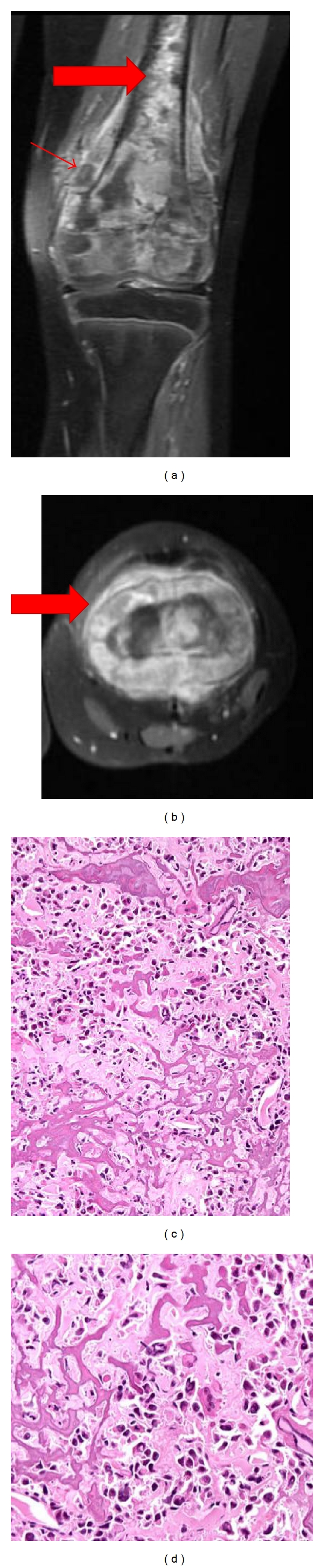
MRI and histological appearance of osteosarcoma. (a) Coronal view MRI reveals diffuse involvement of the distal femur with elevation of the periosteum (small arrow) and diffuse intramedullary involvement (large arrow). (b) Axial view of an MRI reveals elevation of the periosteum due to malignant cells. (c) The classic histological appearance of osteosarcoma. Lace-like pattern osteoid production (small arrow) and malignant cells (large arrow). (d) Higher magnification of (c) to better appreciate the malignant cells.

Osteosarcoma affects the skeleton of patients primarily in regions of rapid bone growth such as the distal femur, proximal tibia and proximal humerus [3].  Figure 1(a) shows a patient MRI with obvious and extensive osteoblastic changes in the metaphysis of the distal femur with elevation of the periosteum and diffuse intramedullary involvement. An MRI of the same femur in axial view reveals the extent of the elevation of the periosteum due to malignant cells (Figure 1(b)). Several studies have identified genetic and epigenetic changes that prevent normal osteoblastic differentiation from mesenchymal progenitor cells [4–7] as a major factor leading to the development of OS [3]. The disease is characterized by rapidly synthesized osteoid that is produced by immature osteoblastic cells [8]. These malignant cells and the immature lace-like osteoid are shown in the histological appearance of OS (Figures 1(c) and 1(d)). The mechanism responsible for OS tumorogenesis is poorly understood with inactivation of tumor suppressor genes and the deregulation of major bone regulatory signaling pathways, such as Wnt, bone morphogenetic protein (BMP), fibroblast growth factor (FGF), and hedgehog signaling all implicated [3].

The majority of OS tumors are heterogeneous, characterized by the various stages of mesenchymal differentiation, each of which demonstrates different levels of gene and protein expression, adding to the complexity of the disease. Despite the heterogeneity within individual tumors and individual patients, mutations in p53 and RB have consistently been identified [3, 9–12]. These proteins are major regulators of the cell cycle, and caution should be used when considering these proteins as therapeutic targets as they may result in many adverse side effects. The terminal differentiation of osteoblasts is controlled by a cascade of regulatory signals such as BMP, Sonic and Indian hedgehog, and core-binding factor *α*-1 (Cbfa1) (also called runt-related gene 2 (Runx2)) [13–15], and therefore, it is unlikely a single treatment targeting a single pathway will be effective [3]. A combination of drugs that target multiple osteoblastic pathways will likely be needed to overcome the drug resistance common in OS [3, 16].

In this paper, we focus on the growing list of available state-of-the-art proteomic techniques and their application to the discovery of OS targets and signaling pathways associated with primary and metastatic OS. These new approaches provide the rationale to shift the focus of OS research away from the single-marker/single-pathway paradigm, to a systems biology approach enabling the analysis of multiple signaling pathways that are involved in primary OS and subsequent metastatic disease [17].

## 2. “Shotgun” Proteomics

Genomic studies provide valuable insight into the regulation of genes involved in the pathogenesis of disease but are limited in their ability to identify multiple protein products derived from individual genes, and it is well established that mRNA levels do not necessarily correlate to the protein level, which is the functional output. Proteomics is a technology for studying protein expression, protein-protein interactions, and posttranslational modifications from complex mixtures of fluids or tissues that reflect the mechanism of disease [17]. Proteomic technologies continue to advance by increasing the scale of protein identification and protein quantification using both label-free and stable isotope labeling techniques [18–24].

Most proteomic techniques involve a protein separation step followed by identification of proteins. The traditional protein separation technique used to study OS includes two-dimensional gel electrophoresis (2DE) followed by matrix-assisted laser desorption/ionization (MALDI) mass spectrometry for protein identification [8, 25–29]. In 2DE, proteins are separated by both isoelectric point (pI) and by molecular weight (MW). The proteins are then extracted from the gel and digested, and each spot is identified by mass spectrometry. This is a tedious process due to the fact each protein must be spotted individually for MALDI analysis, and therefore, only small numbers of proteins have been identified as differentiating in OS. Another disadvantage of this technique is that portions of the proteome such as low-abundant proteins, membrane-associated or bound proteins, and proteins with extremes in pI and MW are rarely identified.

**Figure 2:**
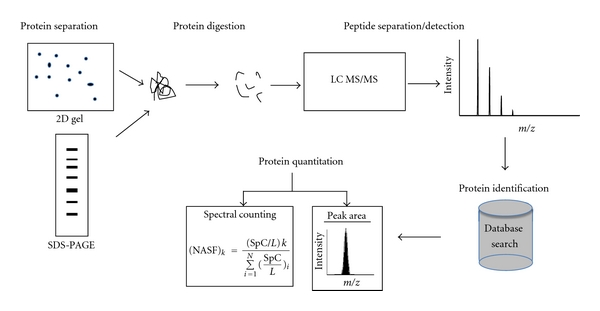
LC MS/MS workflow. Proteins can be separated by 2D or SDS-PAGE prior to mass spectrometry. Proteins are digested into peptides, separated by LC, and identified by mass spectrometry. The mass spectra are searched in a protein database to identify proteins. The proteins are quantified using spectral counting (*k* is a given protein, SpC is the spectral count, *L* is the length of the protein, and *N* is all proteins identified in the gel lane) or peak areas in label-free proteomics.

More recently, proteomic technologies have greatly improved both protein separation and protein identification and can be readily applied to offer deeper insight into OS pathogenesis (Figure 2). One technique is multidimensional protein identification technology (MudPIT), which is a non gel-based approach that uses 2D liquid chromatography (LC) for protein separation prior to identification by mass spectrometry [30–32]. MudPIT works by first digesting proteins into peptides, resolving peptides by 2D-LC, and finally detecting the peptides by MS/MS [31]. The peptides are separated on a pulled microcapillary column packed with two independent chromatography phases, which allows for separation by charge and hydrophobicity to resolve complex peptide mixtures [33]. The peptides are eluted directly into the mass spectrometer for peptide identification, and the proteins are identified by database searching [31, 34]. The coupling of 2D-LC to tandem mass spectrometry (MS/MS) was applied by Link et al. [33] for proteome-wide studies and is able to identify proteins from all subcellular portions of the cell [31].

The development of MudPIT gave rise to other shotgun proteomic technologies, which refers to the direct analysis of complex protein mixtures to rapidly generate a global profile of the protein complement within a sample mixture [35]. Another shotgun approach is a technique called nanocapillary liquid chromatography coupled with tandem mass spectrometry (nanoLC-MS-MS) [36]. Using this method, proteins are resolved by standard one-dimensional SDS-PAGE, and the entire gel lane is excised for trypsin digestion and analyzed by nanoLC-MS-MS. MudPIT and nanoLC-MS-MS have similar protein identification outputs; therefore, both platforms will be useful for OS analysis [36, 37].

## 3. Bioinformatic Analyses

Differentiating proteins between normal bone, OS primary tumors, and metastatic tumors can be accomplished using spectral counting, that is, the number of tandem mass spectra assigned to a given protein [37]. This is a label-free method for protein semiquantitation [38]. Each sample is subjected to individual LC-MS/MS, and the spectral counts of identified proteins are used for direct comparison of samples. Quantitation of proteins is achieved by comparing the number of MS/MS spectra for the same protein between two or more MS/MS analyses. However, it is not always appropriate to use raw spectral counts due to the lack of biological replicates and inability to carry out standard statistical analyses in proteomic studies [39]. Therefore, the data must first be normalized before standard statistical analyses can be performed. Zybailov et al. developed a mathematical normalization technique named the normalized spectral abundance factor (NSAF), which after the natural log of the NSAF value is calculated, transforms the data into a Gaussian/normal distribution allowing for statistical testing using the *t*-test and other more robust parametric methods [39]. We have recently applied label-free shotgun proteomics to study melanoma [37]. Proteins were extracted from formalin-fixed paraffin-embedded (FFPE) tissue from melanocytic nevus and metastatic melanoma. The spectral counts were identified using mass spectrometry, and the data was normalized by NSAF. A total of 390 proteins were found to be differentially regulated between nevus and metastatic melanoma [37]. Another method for protein quantitation involves the alignment of LC MS/MS runs and integrating the peak area for the peptides [40–42].

An alternative to label-free shotgun proteomics for protein quantitation includes stable isotope labeling approaches. These include isotope-coded affinity tag (ICAT) [43–45], stable isotope labeling by amino acids in cell culture (SILAC) [43, 46–50], and isobaric tags for relative and absolute quantification (iTRAQ) [51].

## 4. The Current State of Osteosarcoma Proteomics

**Figure 3:**
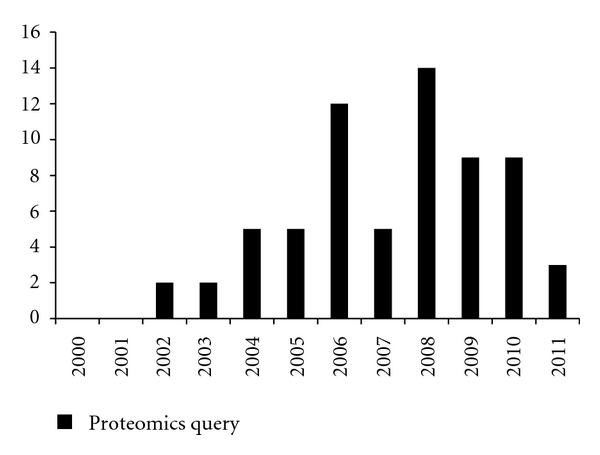
Pubmed Search. The number of research articles identified by Pubmed with the search terms “osteosarcoma” and “proteomics” since 2000. There have been fewer than 70 publications thus far.

The proteomic technologies discussed above have been drastically underappreciated in osteosarcoma research with fewer than 70 analyses published in the last decade (Figure 3), many of which rely on early stage technologies such as 2DE and MALDI MS. Here we briefly review the proteomic studies that have been performed since 2000, highlighting the current prognostic, diagnostic, and predictive biomarkers identified from multiple studies thus far (Table 1; [8, 25–28, 52–60]). A compilation of all differentially expressed proteins identified in these studies can be found in Supplementary Table 1 available online at doi:10.1155/2012/169416. This information has provided useful insight into OS tumorogenesis; however, multiple proteins across multiple signaling pathways that characterize OS are likely to enrich our understanding of the hallmarks of cancer [61, 62] that are unique to OS.

The majority of studies have used differential protein expression to identify new protein markers that can be translated into new diagnostic and therapeutic targets, but they fail to characterize their global contribution to disease pathogenesis and instead focus on single marker/single-pathway identifications leaving out the complexity of multiple pathway interactions. Some examples of proteomics studies which focus mainly on known signaling pathways instead of taking a more global proteomic approach to identify the complexity of protein interactions do exist and are summarized below.

Cates et al. compared protein expression profiles between two clonally related murine OS cell lines with low (K12) and high (K7M2) metastatic potential using two-dimensional difference gel electrophoresis (2D-DIGE) and MALDI [26]. 2D-DIGE allows direct comparison and relative quantification of specific proteins among different samples resolved together on the same gel using different cyanine fluorescent dyes [63, 64]. They identified 9 protein peaks by MALDI-MS with at least a twofold difference in relative ion intensities in K7M2 cells compared to K12 cells. 2D-DIGE and tissue profiling identified 20 proteins, which were uploaded into Ingenuity Pathway Analysis software identifying 95 additional proteins in promoting metastatic disease. Two cytokines, macrophage migration inhibitory factor (MIF: NCBI refseq NM_002415.1) and tumour necrosis factor (TNF: NCBI refseq NP_000585.2), were chosen for further validation. TNF and MIF have potential for use as OS biomarkers and may represent new OS therapeutic targets.

 Y. Li et al. compared primary OS to benign bone tumor samples using 2D gel electrophoresis and the protein spots identified with MALDI-TOF MS [28]. They were able to obtain protein identification from 18 out of the 30 differentiating protein spots from the 2D gel (Table 1). They identified cytoskeleton and microtubule-associated proteins, suggesting they play a role in the tumor cell migration and metastasis that is characteristic of OS [28].

 Zhang et al. took a subcellular comparative approach [56]. They analyzed proteins in the plasma membrane of human OS cell line (MG-63) and human osteoblastic cells (hFOB1.19). Proteins were analyzed through iTRAQ-based quantitative LC/MS/MS [56]. This is the first literature citation of an LC-MS/MS approach in OS; however, they identified only 342 proteins due to their focus on proteins resident in the plasma membrane. Sixty-eight out of the 342 identified proteins were differentially expressed with at least a 1.5-fold difference [56]. Cluster of differentiation 151 (CD151) was chosen for validation using IHC due to its ability to activate the phosphoinositide 3-kinase (PI3K) pathway to promote neovascularization [56].

Engin et al. studied the role of Notch signaling in tumors of mesenchymal origin [65]. Notch has been previously associated with different diseases and cancer [66–69]. A growing body of evidence supports the idea that Notch can function either as an oncogene or a tumor suppressor depending on its expression level [70, 71]. Comparing 7 primary untreated OS samples and 3 posttreatment OS samples with 3 wild-type osteoblasts by RT-PCR, the expression of NOTCH1, Notch ligand JAGGED-1, transcription factors HES-1 and HEY2, direct targets of NOTCH1 signaling, was significantly upregulated in both untreated OS and posttreatment OS compared with wild-type osteoblasts [65]. They also found the osteoblastic-specific transcription factor osterix was upregulated. These data support the notion that gain of Notch function in committed osteoblasts leads to proliferation of an immature osteoblastic population and arrest of osteoblast maturation leading to the development of OS. Notch can also directly inhibit the master osteoblastic transcription factor Runx2. Osteoblast function is regulated, in part, by two specific transcription factors, Runx-2 and osterix [16]. They show Notch signaling occurs downstream of p53 loss of function. In sum, there data suggest that loss of p53 in a committed osteoprogenitor population leads to gain of function of Notch signaling with consequences on cyclins and osterix up-regulation and Runx2 inhibition, perhaps accounting for proliferative and metastatic potential of osteosarcomas [65].

Li et al. used a proteomic analysis approach to understand the role of E2F1 in p53-negative tumor cells [27]. E2F1 is a transcription factor that plays an important role in cell-cycle progression and apoptosis. The overexpression of E2F1 leads to tumor growth suppression, which makes it an interesting therapeutic target; however, because it is a master regulator, the precise mechanisms by which it works need to be identified to avoid major side effects. Li and colleagues studied the effects of E2F1 activity in inducible p53-deficient Saos-2ERE2F1 OS cells using 2DE and MALDI-MS. They identified 33 proteins with 13 proteins coming from genes annotated as cancer related. E2F1 sensitizes cells for apoptosis by inhibition of antiapoptotic survival signals from nuclear factor kappa-B (NF-kappa B) [27]. NF-kappa B mediates inflammatory and antiapoptotic pathways and has been causally linked to OS lung metastasis [16]. Downstream targets of E2F1 include epidermal growth factor (EGF), tumor necrosis factor (TNF), interferon gamma (IFN-gamma), Ca(2+)-induced protein kinase C (PKC), protein kinase A (PKA), the mitogen-activated protein kinase (MAPK) pathway, and the nonreceptor tyrosine kinase SRC [27].

 The c-Jun N-terminal kinase (JNK) signal transduction pathway that is known to play a role in the proliferation, differentiation, and apoptosis of osteoblasts in normal bone has also been studied [72]. These investigators examined JNK expression by immunohistochemistry (IHC) of OS specimens in paraffin-embedded sections from 56 patients with high-grade tumor and 15 patients with low-grade tumor [72]. They assessed the protein levels of two major JNK isoforms (JNK1 and JNK2), their phosphorylated (activated) species, p-JNK; their specific substrate, c-Jun; its phosphorylated form, pc-Jun; c-Jun heterodimeric partner, c-Fos. In addition, these studies also examined the alpha chain of the nascent polypeptide-associated complex (alpha-NAC), an osteoblastic-specific AP-1 coactivator [72]. Positive staining was observed in OS, with higher protein expression observed in patients with high-grade tumors compared with low-grade tumors [72]. The authors conclude that the proteins identified as significantly different between metastatic tumors and normal bone may represent a complex network of signaling pathways, critical for the development of OS.

 Recently, a noninvasive approach for the proteomic profiling of blood from osteosarcoma patients was described [73]. Using surfaced-enhance laser desorption/ionization-time of flight (SELDI-TOF) mass spectrometry. They were able to distinguish a unique protein profile between osteosarcoma patients and patients with benign bone disease. In order to improve biomarker discovery, the researchers developed a method for the depletion of two highly abundant plasma proteins prior to the detection of the low-abundant proteins [73].

## 5. Discussion

OS is the primary bone cancer occurring during the second decade of life and rarely in older adults with Paget's disease. These are times in which there is rapid bone development and turnover. Proteomics has slowly begun to be utilized for the identification of proteins that characterize OS and that may play a role in OS tumorigenesis (Figure 3). The majority of the studies thus far have targeted specific signaling pathways of known association with malignancy. For instance, pathways involved in cell survival and resistance to apoptosis promote lung metastasis and survival after extravasation to the lungs, and the proteins found upregulated by proteomic studies include ezrin, TGF-beta, and apoptotic signaling intermediates [16]. Ezrin has been found to be upregulated in osteosarcoma by several research groups [28, 54, 74, 75]. Phosphorylated Ezrin is thought to play a role in early-stage metastasis by connecting with the target organ site and therefore is thought to be an ineffective treatment strategy as its function is completed once a metastasis is established [2]. However, Ren et al. discovered Ezrin phosphorylation is also present at the leading edge of large metastatic lesions [75]. Targeting Ezrin could be promising for managing lung metastases; however, we should shift the focus from targeting one protein to identifying the complexity of multiple protein interactions to improve the efficacy of treatment. The advancement of proteomics provides the means to study these complex protein networks and identify the multiple pathways involved in tumorogenesis, thus shifting the current research focus of OS away from the single-marker/single-pathway paradigm to a more systems like approach.

In addition to proteomics, new technologies are constantly emerging that provide complementary approaches for the identification of disease-specific biomarkers. Techniques include surface plasmon resonance (SPR) [76–79] and nanowires/nanotube-based field effect transistors (FETs) [19, 24, 80, 81]. SPR detects changes in refractive index of dielectric layer adjacent to the metal film due to adsorption or desorption of molecules on the surface. The optical surface technique monitors changes in a real-time manner. SPR has been used for biomarker discovery in colon cancer [82], ovarian cancer [83], and pancreatic cancer [84].

Nanowires/nanotubes are used to target specific low-molecular-weight proteins. The platform consists of an electrochemical immunosensor, which consists of multiwalled carbon nanotubes for real-time detection of cancer biomarkers in human serum [19]. The technology binds candidate proteins to the functionalized nanowires/nanotubes, leading to a detectable alteration in electrical conductance of the device [19]. Nanowires have been used for biomarker discovery in prostate cancer [24, 81] and myocardial infarction [85].

Although exciting, these biomarker discovery approaches are currently in the proof-of-concept stage and have been used for targeted discovery of low-abundant proteins in human serum and not for *de novo* protein identification. Complementing proteomic-based biomarker platforms with these new approaches may indeed provide the sensitivity and specificity needed for a comprehensive analysis of OS. In sum, we eagerly await the impact of these evolving technologies on the pathophysiology, diagnosis, and treatment of OS.

## Supplementary Material

The Supplemental Material reviews the current state of the proteomic studies performed since 2000 to the present. The table includes references, sample types, proteomic techniques used in the study, the identified proteins that were up- or down-regulated in OS, and the validated biomarkers from each of the proteomic studies.

## Figures and Tables

**Table 1:** Commonly identified proteins from multiple proteomic studies. The first column represents the accession number of the protein, followed by the protein name. The last column contains the author's last name and year of the publication the protein was found to be significantly differentiated with the expression level indicated in parentheses.

Accession	Protein name	References (regulation)
GI: 5453832	150 kDa oxygen-regulated protein (precursor)	Zhang et al. 2009 [52] (down); Spreafico et al. 2006 [53] (up); Niforou et al. 2008 [25]
P08865	40S ribosomal protein SA	Folio et al. 2009 [54] (down); Kang et al. 2006 [55] (down)
P49189	4-Trimethylaminobutyraldehyde dehydrogenase	Guo et al. 2007 [8] (down); Niforou et al. 2008 [25]
P05388	60S acidic ribosomal protein P0	Guo et al. 2007 [8] (up); Kang et al. 2006 [55] (down)
GI: 4916999	78 kDa glucose-regulated protein	Zhang et al. 2009 [52] (down); Niforou et al. 2008 [25]
P60709	Actin, cytoplasmic 1	Hua et al. 2011 [60] (down); Niforou et al. 2008 [25]
P63261	Actin cytoplasmic 2	Folio et al. 2009 [54] (down); Zhang et al. 2010 [56] (down); Niforou et al. 2008 [25]
O95433	Activator of 90 kDa heat shock protein ATPase homolog 1	Guo et al. 2007 [8] (up); Niforou et al. 2008 [25]
P30837	Aldehyde dehydrogenase X, mitochondrial (precursor)	Kang et al. 2006 [55] (up); Niforou et al. 2008 [25]
GI: 127801853	Alkaline phosphatase, liver/bone/kidney	Spreafico et al. 2006 [53] (up); Zhang et al. 2009 [52] (up)
P06733	Alpha enolase	Li et al. 2006 [27] (up); Folio et al. 2009 [54] (down)
P04083	Annexin A1	Cates et al. 2010 [26] (up); Spreafico et al. 2006 [53] (down); Kang et al. 2006 [55] (up); Niforou et al. 2008 [25]
P07355	Annexin A2	Spreafico et al. 2006 [53] (down); Zhang et al. 2010 [56] (up); Niforou et al. 2008 [25]
P08758	Annexin A5	Liu et al. 2009 [57] (down); Niforou et al. 2008 [25]
O95816	BAG-family molecular chaperone	Chang et al. 2008 [58] (down); Niforou et al. 2008 [25]
Q15417	Calponin-3	Guo et al. 2007 [8] (up); Niforou et al. 2008 [25]
P27797	Calreticulin (precursor)	Hua et al. 2011 [60] (up); Zhang et al. 2009 [52] (up); Niforou et al. 2008 [25]
P07339	Cathepsin D precursor	Spreafico et al. 2006 [53] (down); Li et al. 2006 [27] (down)
P12277	Creatine kinase B-type	Spreafico et al. 2006 [53] (up); Niforou et al. 2008 [25]
P06730	Eukaryotic translation initiation factor 4E	Kang et al. 2006 [55] (down); Niforou et al. 2008 [25]
P15311	Ezrin	Li et al. 2010 [28] (up); Folio et al. 2009 [54] (up); Guo et al. 2007 [8] (down)
P52907	F-actin capping protein subunit alpha-1	Kang et al. 2006 [55] (down); Niforou et al. 2008 [25]
P02792	Ferritin light chain	Li et al. 2010 [28] (up); Niforou et al. 2008 [25]
Q02790	FK506-binding protein 4	Guo et al. 2007 [8] (up); Niforou et al. 2008 [25]
P04075	Fructose-bisphosphate aldolase A	Kang et al. 2006 [55] (up); Niforou et al. 2008 [25]
P09972	Fructose-bisphosphate aldolase C	Chang et al. 2008 [58] (up); Kang et al. 2006 [55] (up); Niforou et al. 2008 [25]
P09382	Galectin-1	Zhang et al. 2010 [56] (up); Spreafico et al. 2006 [53] (down); Niforou et al. 2008 [25]
P11142	Heat-shock cognate 71 kDa protein	Li et al. 2006 [27] (up); Niforou et al. 2008 [25]
Q5IST7	Heat-shock 70	Zhao et al. 2010 [59] (down); Liu et al. 2009 [57] (up)
P11142	Heat-shock cognate 71 kDa protein	Li et al. 2006 [27] (up); Niforou et al. 2008 [25]
P31943	Heterogeneous nuclear ribonucleoprotein H	Kang et al. 2006 [55] (up); Li et al. 2006 [27] (up); Niforou et al. 2008 [25]
P61 978	Heterogeneous nuclear ribonucleoprotein K	Li et al. 2006 [27] (down); Niforou et al. 2008 [25]
P14866	Heterogeneous nuclear ribonucleoprotein L	Chang et al. 2008 [58] (up); Kang et al. 2006 [55] (up); Niforou et al. 2008 [25]
Q15181	Inorganic pyrophosphatase	Kang et al. 2006 [55] (down); Niforou et al. 2008 [25]
Q03252	Lamin-B2	Li et al. 2010 [28] (up); Niforou et al. 2008 [25]
P07195	L-Lactate dehydrogenase B chain	Spreafico et al. 2006 [53] (up); Niforou et al. 2008 [25]
P43243	Matrin-3	Li et al. 2006 [27] (down); Niforou et al. 2008 [25]
P19338	Nucleolin	Li et al. 2006 [27] (down); Niforou et al. 2008 [25]
P06748	Nucleophosmin	Zhao et al. 2010 [59] (down); Folio et al. 2009 [54] (down); Niforou et al. 2008 [25]
P35232	Prohibitin	Zhao et al. 2010 [59] (down); Cates et al. 2010 [26] (up); Kang et al. 2006 [55] (down); Niforou et al. 2008 [25]
P25786	Proteasome subunit alpha type 1	Zhang et al. 2009 [52] (down); Li et al. 2006 [27] (down); Niforou et al. 2008 [25]
P14618	Pyruvate kinase isozymes M1/M2	Folio et al. 2009 [54] (down); Guo et al. 2007 [8] (up); Niforou et al. 2008 [25]
Q15459	Splicing factor 3 subunit 1	Chang et al. 2008 [58] (up); Niforou et al. 2008 [25]
P20152	Vimentin	Cates et al. 2010 [57] (up); Li et al. 2010 [28] (up); Zhang et al. 2010 [56] (up); Zhao et al. 2010 [59] (down); Zhang et al. 2009 [52] (down); Kang et al. 2006 [55] (down); Li et al. 2006 [27] (up); Zhao et al. 2006 [86] (up); Niforou et al. 2008 [25]
