# Supplementary material for: Proteomic Technologies for the Study of Osteosarcoma
Source: Sarcoma. 2012 Mar 6;2012:169416. doi: 10.1155/2012/169416 (PMC3329661; doi:10.1155/2012/169416)
Supplement: Supplementary file 1 — The Supplemental Material reviews the current state of the proteomic studies performed since 2000 to the present. The table includes references, sample types, proteomic techniques used in the study, the identified proteins that were up- or down-regulated in OS, and the validated biomarkers from each of the proteomic studies. [file 169416.f1.pdf]

Supplemental Table 1: Differentially expressed proteins identified using proteomic technologies identified by multiple investigators.

Author	Sample Type	Proteomics Techniques	Identified Biomarkers	Validated Biomarkers
Jin et al.<br>2007	Human Serum	2D-DIGE<br>MALDI	**Up-Regulated:**<br>amyloid-related serum protein (SAA)<br>Ceruloplasmin<br>DGKG protein<br>Chain B, human complement component C3<br>alpha-1-B-glycoprotein (GI:69990) + unnamed protein product (GI:1335098)<br>alpha-1-antitrypsin precursor (P01009-00-16-00, P01009-00-03-00, P01009-00-16-00)<br>Complement factor I precursor (EC 3.4.21.45) (C3B/C4B inactivator)<br>ficolin-3 precursor (Collagen/fibrinogen domain-containing protein 3) NL3<br>Plasma retinol-binding protein precursor (PRBP)<br>Chain B, human complement component C3<br>Complement component 7 precursor<br>MHC serum complement factor B<br>Complement factor B<br><br>**Down-Regulated:**<br>Fibronectin 1 isoform 6 preproprotein<br>MTA2 protein<br>Fibronectin 1 isoform 6 preproprotein<br>Fibronectin precursor (FN) (cold-insoluble globulin) (CIG)<br>Inter-alpha-trypsin inhibitor heavy chain H4<br>Fibrinogen gamma<br>Chain A, crystal structure of the Ga module complexed with HSA<br>Gelsolin, isoform A<br>Chain A, crystal structure of the glycosylated five-domain human beta2-glycoprotein I purified from blood plasma<br>Fibrinogen beta chain precursor<br>Fibrinogen gamma chain precursor<br>Fibrinogen gamma chain<br>Transthyretin precursor (Prealbumin) (TBPA) (TTR)<br>Chain E, pentameric crystal structure of human serum amyloid P<br>Chain C, crystal structure of lipid-free human apolipoprotein A-I<br>Human tetranectin, A trimeric plasminogen binding protein with an alpha-helical coiled coil	amyloid-related serum protein (SAA)
Li Z et al.<br>2006	SaOS-2 REF2F1<br>OS cell line	2-DE<br>MALDI	**Up-Regulated:**<br>Glucosidase II alpha subunit (Q14697)<br>Protein disulfide-isomerase A3 precursor (P30101)<br>Alpha enolase (2-phospho-D-glycerate hydrolyase) (P06733)<br>Ubiquitin carboxyl-terminal hydrolase isozyme L1 (P09936)<br>Splicing factor, pre-mRNA splicing factor SF2 (Q07955)<br>Heterogeneous nuclear ribonucleoprotein H (hnRNP H) (P31943)<br>Vimentin (VIM) (P08670)<br>ACTB protein (Q96E67)<br>Tubulin alpha-6 chain (Q9BQE3)<br>Tubulin beta-1 chain (P07473)<br>Rab GDP dissociation inhibitor beta (Rab GDI beta) (GDI-2) (P50395)<br>Histone H2B.1 (Q9UE88)<br>Heat shock cognate 71 kDa protein (HSPA8) (P11142)<br>T-complex protein 1, gamma subunit (TCP-1-gamma) (P49368)<br>Septin 2 (NEDD5 protein homolog) (Q15019)<br><br>**Down-Regulated:**<br>Caldesmon (CDM) (Q05682)	hnRNPH<br>Vimentin<br>hnRNPA1<br>GRP78<br>HDGF<br>GAPDH

			Cathepsin D precursor (CTSD) (P07339)<br>Peroxiredoxin 1 (thioredoxin peroxidase 2) (PRDX1) (Q06830)<br>Clathrin light chain A (LCA) (P09496)<br>HDGF (P51858)<br>Heterogeneous nuclear ribonucleoprotein A3 (hnRNPA3) (P51991)<br>Heterogeneous nuclear ribonucleoproteins A2/B1 (hnRNPA2/B1) (P22626)<br>Heterogeneous nuclear ribonucleoprotein A0 (hnRNPA0) (Q13151)<br>Heterogeneous nuclear ribonucleoprotein A1 (hnRNPA1) (P09651)<br>Heterogeneous nuclear ribonucleoprotein K (hnRNP K) (P61978)<br>Nucleolin (Protein C23) (P19338)<br>2-Oxoisovalerate dehydrogenase beta subunit (P21953)<br>Keratin, type II cytoskeletal 1 (Cytokeratin 1) (P04264)<br>Proteasome subunit alpha type 1 (P25786)<br>78 kDa GRP precursor (GRP78/BIP) (P11021)<br>94 kDa GRP (GRP94) (P14625)<br>Matrin 3 (P43243)<br>RuvB-like 1 (TATA box-binding protein-interacting protein) (Q9Y265)	
Guo et al. 2007	U2OS, IOR/OS9, SaOS-2, hFOB1.19 cell lines	2-DE<br>MALDI	**Up-Regulated:**<br>heterogeneous nuclear ribonucleoprotein K (HNRPK)<br>Pyruvate kinase isozymes M1/M2 (PKM2)<br>FK506-binding protein 4 (FKBP4)<br>Hydroxymethylglutaryl-CoA synthase (HMGCS1)<br>60S acidic ribosomal protein P0 (RPLP0)<br>Adenosylhomocysteinase (AHCY)<br>Activator of 90 kDa heat shock protein ATPase homolog 1 (AHSA1)<br>Stomatin-like protein 2 (STOML2)<br>Calponin-3 (CNN3)<br><br>**Down-Regulated:**<br>Ezrin p81 cytovillin villin-2 (VIL2)<br>Interferon-induced GTP-binding protein Mx1 (MX1)<br>WD repeat protein 1 (WDR1)<br>Dihydropyrimidinase-related protein 2 (DPYSL2)<br>Plastin-3 (PLS3)<br>Coronin-1B (CORO1B)<br>Phosphoacetylglucosamine mutase (PGM3)<br>UDP-N-acetylhexosamine pyrophosphorylase (UAP1)<br>Glutathione trasferase omega-1 (GSTO1)<br>Tryptophanyl-tRNA synthetase (WARS)<br>Desmin (DES)<br>Keratin type II cytoskeletal 8 (KRT8)<br>52 kDa Ro protein (TRIM21)<br>4-trimethylaminobutyraldehyde dehydrogenase (ALDH9A1)<br>Keratin type I cytoskeletal 18 (KRT18)<br>Plasminogen activator inhibitor 2 precursor (SERPINB2)<br>Macrophage capping protein (CAPG)	Stomatin-like protein 2 (STOML2)<br>Activator of 90 kDa heat shock protein ATPase homolog 1 (AHSA1)
Hua et al. 2011	MG-63, hFOB1.19 cell lines	2D-E<br>LC-MS/MS	Up-Regulated:<br>NDRG1<br>HLA-B27<br>NDUFS3<br>CALR<br><br>Down-Regulated:	NDRG1

			ACTB	
Cates et al. 2010	K7M2, K12 cell lines	2-DE<br>MALDI	**Up-Regulated:**<br>Vimentin<br>Endoplasmin<br>Chloride intracellular channel 4<br>Transaldolase<br>Annexin A1<br>Ubiquitin carboxyl-terminal hydrolase 5<br>Prohibitin<br>Proteasome activator complex subunit 2<br>Eukaryotic initiation factor 4A-I<br>Voltage-dependent anion-selective channel protein 1<br>Gelsolin<br><br>**Down-Regulated:**<br>Advillin<br>Elongation factor Tu<br>Farnesyl pyrophosphate synthetase<br>Acyl-coenzyme A thioesterase 2<br>V-type proton ATPase subunit B, brain isoform	macrophage migration inhibitory factor (MIF)<br>tumour necrosis factor (TNF)
Zhang et al. 2010	MG-63, hFOB1.19 cell lines	iTRAQ<br>LC-MS/MS	**Up-Regulated:**<br>IPI00413696.5<br>IPI00827658.1<br>IPI00895803.1<br>ABCC1<br>ACTN1<br>ANPEP<br>ANXA2<br>ATP2B4<br>BST2<br>CD151<br>CD9<br>CD99<br>FER1L3<br>LGALS1<br>LGALS3<br>LITAF<br>MYADM<br>PLEC1<br>S100A10<br>SCRIB<br>SLC29A1<br>SLC3A2<br>STOM<br>VIM<br><br>**Down-Regulated:**<br>IPI00101961.2<br>IPI00413958.4<br>IPI00478565.2<br>IPI00867588.1<br>IPI00871932.1<br>IPI00872684.1	CD151

			IPI00872814.1<br>IPI00873444.1<br>IPI00879810.1<br>IPI00894325.1<br>IPI00894498.1<br>ACTG1<br>B2M<br>CTNNA1<br>CTNNB1<br>CTNND1<br>FAM62A<br>FGR<br>H1ST2H4B<br>H2AFX<br>H2BFS<br>HIST1H2AD<br>HIST1H2BL<br>HIST1H3J<br>HIST2H3A<br>HIST2H4B<br>HSP90B1<br>INTS8<br>IQGAP1<br>ITGA5<br>MARCKS<br>MARCKSL1<br>MYH9<br>MYL6<br>PVRL2<br>RALA<br>RALB<br>RAP1B<br>SLC16A3<br>STX8<br>TUBA1B<br>TUBB2B<br>XTP3TPATP1	
Zhoa et al. 2010	MG-63 cell line	2-DE<br>MALDI<br>WB	**Up-Regulated:**<br>DNA-Damage-inducible transcript<br>Discoidin<br>DNA polymerase zeta<br>HCV-2<br>Bullous pemphigoid antigen 1 isoforms<br>Growth-regulating protein<br>Cyclic nucleotide hosphodiesterase<br><br>**Down-Regulated:**<br>Mannose 6-phosphate receptor<br>ARMC8 protein<br>HERV-K_1q23.3<br>GTPase activating Rap<br>Nucleophosmin<br>Glutamate dehydrogenase	Nucleophosmin<br>Prohibitin<br>Vimetin

			TCP-1-beta<br>Pyrophosphatase 1<br>TER ATPase<br>Vimentin<br>Prohibitin<br>Mutant beta-globin<br>PROM2 protein<br>Heat-shock 70	
Li Y et al. 2010	Human biopsies	2-DE<br>MALDI	**Up-Regulated:**<br>Lamin B2 (LMNB2)<br>Vimentin (VIM)<br>Coatomer protein complex, subunit ε (COPE)<br>Actin-γ1 (ACTG1)<br>Zinc finger protein 133 (ZNF133)<br>Tubulin-α1c (TUBA1C)<br>Ferritin, light polypeptide (FTL)<br>Myosin, light chain 6, alkali, smooth muscle and non-muscle (MYL6)<br>Ezrin (EZR)<br>Transferrin (TF)<br>α1-Antitrypsin (SERPINA1)<br>Chaperonin containing TCP1, subunit 8 (θ) (CCT8)<br><br>**Down-Regulated:**<br>Adenylate cyclase 1 (brain) (ADCY1)<br>Actin-β (ACTB)<br>Tubulin-β (TUBB)<br>ATP synthase, mitochondrial F1 complex, β polypeptide (ATP5B)<br>Reticulocalbin 3, EF-hand calcium-binding domain (RCN3)<br>Tyrosine 3-monooxygenase/tryptophan 5-monooxygenase activation protein, ζ polypeptide (YWHAZ)	Zinc finger protein 133 (ZNF133)<br>Tubulin-α1c (TUBA1C)
Zhang YK et al. 2009	SaOS-2 cell ine	2-DE<br>MALDI	**Up-Regulated:**<br>ALPL protein variant<br>Alkaline phosphatase<br>Alkaline phosphatase, liver/bone/kidney<br>Tissue nonspecific alkaline phosphatase precursor<br>Alpha-internexin (alpha-Inx) (66kDa neurofilament protein)<br>Calreticulin precursor (homo sapiens)<br>Keratin 10<br>Phosphofructokinase, platelet-type<br>hCG1644186 (homo sapiens)<br><br>**Down-Regulated:**<br>150kDa oxygen regulated protein precursor<br>Stress-70 protein, mitochondrial precursor (75kDa glucose-related protein)<br>78kDa glucose-regulated protein<br>BIP<br>Heat shock 70kDa protein 8 isoform 1<br>Mitochondrial matrix protein<br>Chaperonin (homo sapiens)<br>Chaperonin (HSP60)<br>Nucleobindin-1 precursor (CALNUC)<br>Vimentin<br>Actin, gamma-1 propeptide (homo sapiens)<br>Heat shock 60kDa protein 1 (chaperonin)	N/A

			Proteasome subunit C2<br>Proteasome subunit alpha type 1<br>Ubiquitin carboxy-terminal hydrolase L1<br>hCG1647962, isoform CRA_b<br>Ras-associated protein Rap1<br>chain B, solution Nmr structure of protein dynein light chain 2a	
Folio et al. 2009	Human Biopsies	2D-DIGE<br>nano-LC-ESI-MS/MS	**Up-Regulated:**<br>Heat shock protein beta 6 (HSPB6 human)<br>Heme binding protein 1 (HEBP1 human)<br>Ubiquitin carboxyl terminal hydrolase isozyme L1 (UCHL)<br>Ezrin p81 (EZRI human)<br>LIM and SH3 domain protein 1 (LASP1 human)<br>Thioredoxin reductase 1 (TRXR1 human)<br>Peroxirredoxin 6 (PRDX6 human)<br>Alpha crystallin beta chain (CRYAB human)<br>Septin 11 (SEP11 human)<br><br>**Down-Regulated:**<br>40S ribosomal protein SA (RSSA human)<br>Nucleophosmin (NPM human)<br>Heat shock cognate 71KDa protein (HSP7C human)<br>Alpha enolase (ENOA human)<br>Fascin (FSCN 1 human)<br>Pyruvate kinase isozymes M1/M2 (KPYM human)<br>Actin citoplasmic 2 (ACTG human)	alpha-crystallin B chain (CRYAB)<br>Ezrin (EZR1).
Liu et al. 2009	SaOS-2 cell line, human OS/OB primary cultures (cranial bone, 27 week old abortus)	2-DE<br>MALDI	**Up-Regulated:**<br>Heat shock protein 70<br>Mthsp75<br>ATP synthase<br>UQCRC1<br>Ras-related nuclear protein<br>Actin capping protein<br>UCH-L1<br>PRDX4<br><br>**Down-Regulated:**<br>Pyruvate dehydrogenase E1<br>Annexin V<br>Prohibitin	UQCRC1<br>UCH-L1<br>PRDX4
Chang et al. 2008	U2OS, Hep-G2, THP-1 cell lines	2-DE<br>MALDI	**Up-Regulated:**<br>Fructose-bisphosphate aldolase C<br>Prolyl 4-hydroxylase alpha-2<br>Gluetamate-cysteine ligase catalytic subunit<br>Indoleamine 2,3-dioxygenase<br>Endoplasmic reticulum protein<br>Endoribonuclease<br>Cystinyl aminopeptidase<br>Calsequestrin-1 precursor<br>Plastin-2<br>Heterogeneous nuclear ribonucleoprotein L<br>Splicing factor 2 subunit 1<br>Metaphase chromosomal protein 1<br>Clq-related factor	Matrix metalloproteinase-2 (MMP-2)<br>endothelial protein C receptor (EPCR)

			Syntaxin 1B<br>Syntaxin-12<br>SH2 domain protein C2<br>Fibronectin-like domain-containing 1 eucine-rich transmembrane protein 1<br>CDC45-related protein<br>Copine VII<br><br>**Down-Regulated:**<br>Pyruvate kinase isozymes<br>CD85k antigen<br>Endothelial protein C receptor (EPCR)<br>Hydroxyindole O-methyltransferase<br>BAG-family molecular chaperone regulator-2<br>Matrix metalloproteinase-2 (MMP-2)<br>U-PAR<br>Tropomyosin 2<br>DEAD-box protein 25<br>Retinoblastoma-binding protein 1<br>Yes-associated protein<br>Stress-induced-phosphoprotein 1<br>Glutathione S-transferase Mu<br>Syntaxin-binding protein 1<br>Clq-related factor<br>Syntaxin 1B<br>Ras-related protein<br>Pyruvate dehydrogenase<br>Golgi phosphoprotein 2	
Niforou et al. 2008	U2OS cell line	2-DE<br>MALDI<br>MALDI-MS/MS	Chloride intracellular channel protein 1, NCC27<br>Phosphatidylethanolamine-binding protein 1, Raf kinase inhibitor protein<br>Heat-shock protein HSP 90-beta<br>T-complex protein 1 subunit epsilon<br>T-complex protein 1 subunit theta, TCP-1-theta<br>T-complex protein 1 subunit beta, CCT-beta<br>FK506-binding protein 4<br>Proteasome activator complex subunit 3, Ki nuclear autoantigen, PA28g<br>Proteasome activator complex subunit 1, PA28alpha<br>Proteasome activator complex subunit 2, PA28beta<br>Pyridoxal kinase<br>Acyl-protein thioesterase 1<br>Phosphoglycerate kinase 1<br>Fructose-bisphosphate aldolase A<br>L-lactate dehydrogenase B chain<br>Adenine phosphoribosyltransferase<br>Glutathione S-transferase P<br>Fructose-bisphosphate aldolase C<br>Thioredoxin<br>S-formylglutathione hydrolase, Esterase D<br>C-1-tetrahydrofolate synthase, cytoplasmic<br>Inosine-5'-monophosphate dehydrogenase 2<br>Creatine kinase B-type<br>Acylamino-acid-releasing enzyme, Acyl-peptide hydrolase<br>Alcohol dehydrogenase [NADP+]<br>Pyruvate kinase isozymes M1/M2	FK506-binding protein 4 (FKBP4)<br>Src substrate cortactin, Oncogene EMS1 (SRC8)<br>26S proteasome non-ATPase regulatory subunit 10, Gankyrin (PSD10)<br>Far upstream element-binding protein 1, FUSE-binding protein 1, FBP (FUBP1)<br>Protein DJ-1, Oncogene DJ1 (PARK7)<br>Nucleophosmin (NPM)<br>Protein disulfide-isomerase [Precursor] (PDIA1)<br>150 kDa oxygen-regulated protein [Precursor] (OXRP)<br>Protein SET (SET)<br>Translationally-controlled tumor protein, Fortilin (TCTP)<br>Stress-70 protein, mitochondrial [Precursor], Mortalin (GRP75)

			Phosphoglycerate mutase 1, Phosphoglycerate mutase isozyme B<br>Cytosol aminopeptidase<br>Flavin reductase, Biliverdin reductase B<br>Peroxiredoxin-2<br>4-trimethylaminobutyraldehyde dehydrogenase, Gamma-aminobutyraldehyde dehydrogenase<br>GMP synthase [glutamine-hydrolyzing]<br>Galactokinase<br>26S proteasome non-ATPase regulatory subunit 4<br>Peroxiredoxin-1<br>Peroxiredoxin-4<br>Platelet-activating factor acetylhydrolase IB subunit gamma<br>Inorganic pyrophosphatase<br>Phosphomannomutase 1, PMM 1<br>Histidine triad nucleotide-binding protein 2, HINT-2<br>Calpain small subunit 1, Calcium-dependent protease small subunit 1<br>Peroxiredoxin-6, 1-Cys peroxiredoxin, Antioxidant protein 2<br>Alpha-enolase, C-myc promoter-binding protein, MBP-1<br>Src substrate cortactin, Oncogene EMS1<br>Thioredoxin-like protein 2, PKC-theta-interacting protein<br>14-3-3 protein sigma<br>Profilin-2<br>14-3-3 protein zeta/delta<br>COP9 signalosome complex subunit 4<br>Ubiquilin-1, hPLIC-1<br>Rho GDP-dissociation inhibitor 1, Rho GDI 1<br>Chloride intracellular channel protein 4, Intracellular chloride ion channel protein p64H1<br>Elongation factor 2<br>Ubiquitin<br>G-rich sequence factor 1, GRSF-1<br>Heterogeneous nuclear ribonucleoprotein C<br>40S ribosomal protein S12<br>Eukaryotic translation initiation factor 3 subunit 2, eIF3i, TRIP-1<br>Heterogeneous nuclear ribonucleoprotein C [Fragment]<br>Ferritin light chain<br>Tubulin beta chain<br>Alpha-actinin-1<br>Actin, cytoplasmic 2, Gamma-actin<br>Drebrin<br>Fatty acid-binding protein, epidermal, Psoriasis-associated fatty acid-binding protein homolog<br>Uncharacterized protein C11orf73<br>Tether containing UBX domain for GLUT4, Alveolar soft part sarcoma locus<br>Gamma-enolase<br>NG,NG-dimethylarginine dimethylaminohydrolase 1, DDAH-1<br>Activator of 90 kDa heat shock protein ATPase homolog 1, p38, AHA1<br>26S proteasome non-ATPase regulatory subunit 10, Gankyrin<br>Annexin A5<br>Cofilin-1, p18<br>LIM and SH3 domain protein 1, LASP-1<br>Stress-induced-phosphoprotein 1, STI1, Hsc70/Hsp90-organizing protein<br>Four and a half LIM domains protein 2, Skeletal muscle LIM-protein 3<br>Heat-shock protein beta-1, Heat-shock 27 kDa protein<br>Heat-shock cognate 71 kDa protein<br>Nuclear autoantigenic sperm protein, NASP	

			Prefoldin subunit 3, Von Hippel-Lindau-binding protein 1, VBP-1<br>26S protease regulatory subunit S10B<br>26S protease regulatory subunit 6A, Tat-binding protein 1<br>Proteasome subunit alpha type 7<br>Glyceraldehyde-3-phosphate dehydrogenase, GAPDH<br>Nucleoside diphosphate kinase B<br>Proteasome subunit alpha type 1<br>Proteasome subunit beta type 4 [Precursor]<br>Glutathione synthetase<br>RuvB-like 1, TIP49a<br>Far upstream element-binding protein 1, FUSE-binding protein 1, FBP<br>Protein DJ-1, Oncogene DJ1<br>Far upstream element-binding protein 2, KSRP, FUSE-binding protein 2<br>Splicing factor 1, Transcription factor ZFM1<br>Heterogeneous nuclear ribonucleoprotein R<br>Heterogeneous nuclear ribonucleoproteins C1/C2<br>Heterogeneous nuclear ribonucleoprotein L<br>Heterogeneous nuclear ribonucleoprotein M, hnRNP M<br>Eukaryotic translation initiation factor 5A-1, eIF-5A-1<br>Heterogeneous nuclear ribonucleoprotein D0, AUF1<br>Poly(rC)-binding protein 1, Alpha-CP1<br>Poly(rC)-binding protein 2, Alpha-CP2<br>Plasminogen activator inhibitor 1 RNA-binding protein<br>Polyadenylate-binding protein 1<br>Nucleolin<br>Acidic leucine-rich nuclear phosphoprotein 32 family member A, Mapmodulin, Acidic nuclear phosphoprotein pp32<br>Hepatoma-derived growth factor<br>Major vault protein<br>Abhydrolase domain-containing protein 14B<br>26S proteasome non-ATPase regulatory subunit 9<br>40S ribosomal protein SA, 34/67 kDa laminin receptor<br>Gelsolin [Precursor], ADF<br>Vinculin<br>Tropomyosin beta chain<br>Actin, cytoplasmic 1<br>Keratin, type I cytoskeletal 18, Cytokeratin-18<br>Vimentin<br>Keratin, type I cytoskeletal 10<br>Keratin, type I cytoskeletal 9, Keratin-9<br>F-actin capping protein subunit beta<br>F-actin capping protein subunit alpha-1<br>Calponin-3<br>Nucleophosmin<br>Dynactin subunit 2<br>Histidine triad nucleotide-binding protein 1, Protein kinase C inhibitor 1<br>Mitotic spindle assembly checkpoint protein MAD1<br>Lamin-B2<br>Protein disulfide-isomerase A6 [Precursor]<br>Endoplasmin [Precursor], Heat-shock protein 90 kDa beta member 1, GRP94<br>Calreticulin [Precursor]<br>Superoxide dismutase [Cu-Zn]<br>Protein disulfide-isomerase A3 [Precursor], ERp60	

			Endoplasmic reticulum protein ERp29 [Precursor], ERp28<br>Peptidyl-prolyl cis-trans isomerase A, Cyclophilin A<br>Protein disulfide-isomerase [Precursor]<br>Serpin H1 [Precursor], Collagen-binding protein, Colligin<br>Thioredoxin domain-containing protein 12 [Precursor]<br>Reticulocalbin-1 [Precursor]<br>Elongation factor 1-beta, EF-1-beta<br>Reticulocalbin-2 [Precursor], E6-binding protein<br>Glucosidase 2 subunit beta [Precursor]<br>150 kDa oxygen-regulated protein [Precursor]<br>Neutral alpha-glucosidase AB [Precursor]<br>Calumenin [Precursor]<br>Transitional endoplasmic reticulum ATPase, VCP, Valosin-containing protein<br>Protein SET<br>Nucleoporin 50 kDa<br>Serum albumin [Fragment]<br>Hemiferrin<br>Translationally-controlled tumor protein, Fortilin<br>78 kDa glucose-regulated protein [Precursor], GRP 78, BiP<br>Zyxin<br>14 kDa phosphohistidine phosphatase<br>Growth factor receptor-bound protein 2, Adapter protein GRB2<br>Annexin A2<br>Caprin-1, p137GPI<br>Galectin-1<br>Ras GTPase-activating protein-binding protein 1, HDH-VIII, G3BP-1<br>60 kDa heat shock protein, mitochondrial [Precursor]<br>Aldehyde dehydrogenase, mitochondrial<br>NADH dehydrogenase [ubiquinone] flavoprotein 2, mitochondrial [Precursor]<br>3-mercaptopyruvate sulfurtransferase<br>NADH-ubiquinone oxidoreductase 75 kDa subunit, mitochondrial [Precursor]<br>Enoyl-CoA hydratase, mitochondrial [Precursor]<br>Aldehyde dehydrogenase X, mitochondrial [Precursor]<br>Ubiquinol-cytochrome-c reductase complex core protein 1, mitochondrial [Precursor], Core I protein<br>3-hydroxyisobutyrate dehydrogenase, mitochondrial [Precursor]<br>Pyrroline-5-carboxylate reductase 1<br>7,8-dihydro-8-oxoguanine triphosphatase, 8-oxo-dGTPase<br>Dihydrolipoyllysine-residue succinyltransferase component of 2-oxoglutarate dehydrogenase complex, mitochondrial [Precursor], E2K<br>Isocitrate dehydrogenase [NAD] subunit alpha, mitochondrial [Precursor], NAD(+)-specific ICDH<br>Succinyl-CoA:3-ketoacid-coenzyme A transferase 1, mitochondrial [Precursor], Scot-S<br>Aconitate hydratase, mitochondrial [Precursor]<br>40 kDa peptidyl-prolyl cis-trans isomerase, Cyclophilin-40, CYP-40<br>ATP synthase subunit beta, mitochondrial [Precursor]<br>Mitochondrial inner membrane protein, Mitofilin<br>Profilin-1<br>Ubiquitin-conjugating enzyme E2 N, Ubc13<br>Single-stranded DNA-binding protein, mitochondrial [Precursor]<br>14-3-3 protein epsilon, 14-3-3E<br>D-3-phosphoglycerate dehydrogenase<br>Stress-70 protein, mitochondrial [Precursor], Mortalin<br>Prohibitin<br>Heat-shock 70 kDa protein 1	

			10 kDa heat-shock protein, mitochondrial<br>Nascent polypeptide-associated complex subunit alpha, NAC-alpha<br>Nucleoside diphosphate kinase A, nm23-H1<br>Deoxyuridine 5'-triphosphate nucleotidohydrolase, mitochondrial [Precursor], dUTP pyrophosphatase<br>Complement component 1 Q subcomponent-binding protein, mitochondrial [Precursor], Glycoprotein gC1qBP, p33<br>Heat-shock protein 105 kDa<br>Protein C14orf166<br>Elongation factor 1-delta<br>DnaJ homolog subfamily C member 9<br>Replication protein A 32 kDa subunit, RP-A<br>DNA-directed RNA polymerase I 40 kDa polypeptide<br>Probable ATP-dependent RNA helicase DDX5, RNA helicase p68<br>Proliferating cell nuclear antigen<br>Ran-specific GTPase-activating protein, RanBP1<br>Transcription intermediary factor 1-beta, KRAB-associated protein 1, KAP-1<br>Cleavage and polyadenylation specificity factor 5<br>Eukaryotic translation initiation factor 4E<br>Heterogeneous nuclear ribonucleoproteins A2/B1<br>Heterogeneous nuclear ribonucleoprotein H3<br>Heterogeneous nuclear ribonucleoprotein H<br>Cleavage stimulation factor 64 kDa subunit, CstF-64<br>Heterogeneous nuclear ribonucleoprotein F, hnRNP F<br>Heterogeneous nuclear ribonucleoprotein H'<br>Heterogeneous nuclear ribonucleoprotein K, hnRNP K<br>Splicing factor, arginine/serine-rich 1, ASF-1<br>Splicing factor 3A subunit 3, SF3a60<br>Splicing factor 3 subunit 1, SF3a120<br>Heterogeneous nuclear ribonucleoprotein D-like, hnHNRP-DL, JKT41-binding protein<br>Splicing factor, proline- and glutamine-rich, PSF<br>Nuclease sensitive element-binding protein 1, Y-box-binding protein 1, YB-1<br>Breast carcinoma amplified sequence 2<br>Matrin-3<br>High mobility group protein B1<br>Nucleoporin p54<br>NSFL1 cofactor p47<br>Lamin-A/C<br>Annexin A1<br>BAG family molecular chaperone regulator 2, BAG-2<br>ATP synthase D chain, mitochondrial<br>Ubiquitin-activating enzyme E1<br>Triosephosphate isomerase<br>Glyoxylate reductase/hydroxypyruvate reductase<br>Enhancer of rudimentary homolog<br>Annexin A6, P68<br>Myosin light polypeptide 6, Smooth muscle and nonmuscle myosin light chain alkali 6<br>Tubulin alpha-1B chain<br>Tubulin beta-2C chain<br>UPF0160 protein MYG1<br>Transgelin-2<br>Hypothetical protein DKFZp686J1372<br>Nuclear autoantigenic sperm protein<br>Glyoxalase domain-containing protein 4	

			Tubulin alpha-1C chain<br>TRM112-like protein	
Kang et al. 2006	U2OS cell line	2-DE<br>MALDI	**Up-Regulated:**<br>Amphiphysin II<br>A-kinase anchor protein 5<br>Heterogeneous nuclear ribonucleoprotein L<br>Leucine-rich B7 protein<br>Cytochrome c-type heme lyase<br>General transcription factor III<br>atlastin<br>endoplasmic reticulum protein ERp29 precursor<br>Pulmonary surfactant- associated protein D precursor<br>LIM domains containing protein 1<br>Tropomyosin 1 alpha chain<br>Caspase-3 precursor<br>Spry-3<br>Proteasome subunit beta type 3<br>Visinin-like protein 3<br>Retinoic acid- and interferon -inducible 58 kDa protein<br>lysine carboxypeptidase<br>glucose-regulated protein precursor<br>neurogenic differentiation factor 2<br>Disulfide isomerase ER-60<br>Hepatocellular carcinoma- associated antigen 66<br>Glutamate receptor 2<br>Rab GDP dissociation inhibitor beta<br>Pre-B-cell leukemia transcription factor-2<br>Annexin A1<br>Hypothetical protein<br>Fibulin-5 precursor<br>Phosphoglycerate mutase 1<br>fructose-bisphosphate aldolase C<br>Ubiquinol-cytochrome-creductase complex core protein I<br>Isopentenyl pyrophosphate isomerase 2<br>Phosphoglucomutase-like protein 5<br>Fructose-bisphosphate aldolase A<br>TCP-1-theta<br>Aldose reductase<br>Aldehyde dehydrogenase X, mitochondrial precursor<br>transmembrane protease<br>spindlin-like protein 3<br>Matrilin-3 precursor<br>PI3-kinase p85-beta subunit<br>G protein-coupled receptor kinase 7<br>Heterogeneous nuclear ribonucleoprotein H<br>Inhibitor of growth protein 4<br>squamous cell carcinoma antigen 1<br>NY–REN-43 antigen<br>Ras-related protein Rab-3B<br>Zeta-sarcoglycan<br>IFIT-2 (interferon-induced 54 kDa protein<br>Extracellular matrix protein 1 precursor<br>gamma-soluble NSF attachment protein	EGFR<br>MMP-2<br>Caspase-3<br>Annexin I<br>ErK 2

			Mdm4 protein<br>cyclin H<br>Breast carcinoma amplified sequence 1<br>Melanoma-associated antigen G1<br>Cofilin-1<br>SH3-binding protein CBL-B<br>Erk 2<br>Dihydroxyacetone phosphate acyltransferase<br>Flotillin-1<br>Villin 1<br>Protein FAM49B<br>CDC47 (MCM7)<br><br>**Down-Regulated:**<br>Ribulose-5-phosphate- epimerase<br>epsilon-coat protein<br>MMP-2<br>Protein-glutamine gamma- glutamyltransferase 6<br>Peroxiredoxin 2<br>Eukaryotic translation initiation factor 5A<br>Nucleoporin Nup107<br>Vimentin<br>Annexin A3<br>F-actin capping protein alpha-1 subunit<br>Glutathione S-transferase Mu 2<br>Pyridoxal phosphate phosphatase<br>Ubiquitin thiolesterase 24<br>Heterogeneous nuclear ribonucleoproteins C1/C2<br>Heat-shock 27 kDa protein<br>Thyroid receptor interacting protein 3<br>Nucleoside diphosphate kinase A<br>Heat-shock protein beta-8<br>Lamin A/C<br>Tropomyosin 4<br>Phosducin-like protein<br>ATP synthase D chain, mitochondrial<br>Glutathione S-transferase A5–5<br>Eukaryotic translation initiation factor 4E<br>PPAR-beta<br>phenol-sulfating phenol sulfotransferase 1<br>Transcription factor E4TF1–60<br>Aldehyde reductase<br>Inorganic pyrophosphatase<br>Tyr-DNA phosphodiesterase 1<br>60S acidic ribosomal protein P0<br>Heat-shock protein beta-7<br>DNA-repair protein XRCC1<br>Septin-5<br>WDR43 protein<br>Tropomodulin 3<br>Aspartate aminotransferase<br>Enolase 3<br>Prohibitin	

			40S ribosomal protein SA<br>MHC class I antigen<br>Phosphatidylinositol transfer protein alpha isoform<br>Tropomyosin beta chain<br>Fragile X mental retardation 1 protein<br>Potassium voltage-gated channel subfamily A member 1<br>Ubiquitin thiolesterase 11<br>Epidermal growth factor receptor<br>p53 and DNA damage- regulated protein<br>acidic nucleoplasmic DNA- binding protein 1<br>X-linked protein STS1769<br>Kelch-like ECH-associated protein 1<br>Heat shock 70 kDa protein 4<br>Protein phosphatase 2C beta isoform<br>ATP-dependent RNA helicase DDX1<br>TCP-1-zeta	
Spreafico et al. 2006	Human mature OB (E1P2) cultures<br>SaOS-2 cell lines	2-DE<br>MALDI	**Up-Regulated:**<br>Pyruvate kinase M1<br>L-lactate dehydrogenase B chain<br>Triose phosphate isomerase 1<br>Creatine kinase B chain<br>Heat shock protein 90<br>150 kDa oxygen-regulated protein<br>Retinoblastoma-binding protein 4<br>Alkaline phosphatase<br><br>**Down-Regulated:**<br>Galectin 1<br>Annexin I<br>Annexin II<br>Osteonectin<br>Cathepsin D<br>Tropomyosin 3<br>Heat shock protein 27<br>Superoxide dismutase<br>Glutamate dehydrogenase 1<br>UMP-CMP kinase<br>Enoyl-CoA hydratase-like protein	Pyruvate kinase M1<br>L-lactate dehydrogenase B chain<br>Triose phosphate isomerase 1<br>Creatine kinase B chain<br>Heat shock protein 90<br>150 kDa oxygen-regulated protein<br>Retinoblastoma-binding protein 4<br>Alkaline phosphatase<br>Galectin 1<br>Annexin I<br>Annexin II<br>Osteonectin<br>Cathepsin D<br>Tropomyosin 3<br>Heat shock protein 27<br>Superoxide dismutase<br>Glutamate dehydrogenase 1<br>UMP-CMP kinase<br>Enoyl-CoA hydratase-like protein
Zhao et al. 2006	MG-63 cell line	2-DE	**Up-Regulated:**<br>MHC class II antigen<br>Interferon-stimulated gene factor 3d<br>Hypothetical protein DKFZp43M2221.1<br>8-hydroxy-guanine glocosylase homolog ogg1<br>Vimentin<br><br>**Down-Regulated:**<br>hnRNP A2/B1<br>actin	
Zhao et al. 2006	MG-63 cell line	2-DE<br>MALDI	**Up-Regulated:**<br>(MHC) class I1 antigen<br>interferonstimulated gene factor 3 alpha 91/84 kDa protein	N/A

			hypothetical protein DKFZp434M2221.1<br>8-hydroxy-guanine glycosylase homolog ogg1<br>Solute carrier family 25, member 16; Rho GTPase activating protein 19<br>Vimentin<br><br>**Down-Regulated:**<br>hnRNP A2<br>hnRNP B1	
